# Enhanced Detection of Residual Breast Cancer Post-Excisional Biopsy: Comparative Analysis of Contrast-Enhanced MRI with and Without Diffusion-Weighted Imaging

**DOI:** 10.3390/tomography11010010

**Published:** 2025-01-20

**Authors:** Han Song Mun, Bong Joo Kang, Sung Hun Kim, Ga Eun Park

**Affiliations:** Department of Radiology, Seoul St. Mary’s Hospital, College of Medicine, The Catholic University of Korea, Seoul 06591, Republic of Korea; im_hsm@catholic.ac.kr (H.S.M.); rad-ksh@catholic.ac.kr (S.H.K.); hoonhoony@naver.com (G.E.P.)

**Keywords:** breast cancer, excisional biopsy, MRI, residual disease, breast imaging

## Abstract

Objectives: To evaluate the effectiveness of breast MRI, including diffusion-weighted imaging (DWI), in detecting residual lesions in patients with malignancy after excisional biopsy. Methods: From January 2018 to December 2023, 3T breast MRI was performed to assess lesion morphology, residual size, and enhancement kinetics. The apparent diffusion coefficient (ADC) values were measured, and the diagnostic outcomes of CE-MRI, CE-MRI with DWI, mammography (MG), and ultrasound (US) were compared with clinical and histopathological data. Results: A total of 152 lesions were analyzed, with 36.2% showing residual malignancy. Both CE-MRI and CE-MRI with DWI effectively identified residual lesions, with significant differences in morphology, size, kinetic patterns, and ADC values (all *p* < 0.001). CE-MRI with DWI showed a sensitivity of 90.9% and an NPV of 93.6%, compared with 89.1% sensitivity and 92.2% NPV for CE-MRI alone. Sensitivities for MG and US were 57.1% and 38.7%, with NPVs of 64.7% and 59.6%, respectively. Diagnostic accuracy was highest for CE-MRI with DWI (80.9%), followed by CE-MRI (79.0%), MG (60.3%), and US (59.7%). The AUC for CE-MRI with DWI (0.831) was slightly higher than CE-MRI alone (0.811), though not significant (*p* = 0.095). AUCs for MG and US were lower at 0.623 and 0.563, with no significant difference between MG and US (*p* = 0.234). Conclusions: CE-MRI with DWI and CE-MRI alone were comparable and demonstrated excellent performance in discriminating between women with and without residual disease. Integrating CE-MRI with DWI could become a standard protocol for patients with suspected residual malignancy after excisional biopsy.

## 1. Introduction

Dynamic contrast-enhanced MRI (CE-MRI) of the breast has become a critical tool for patients with known or suspected breast cancer, consistently demonstrating high sensitivity between 89% to 100%, albeit with more variable specificity ranging from 26% to 91% [1,2,3,4].

After an excisional biopsy of breast carcinoma, residual tumors remain in 32% to 63% of patients [5,6]. The risk of local recurrence increases if a residual tumor is present or if the biopsy margins are close [7,8,9]. Detecting residual disease and determining its extent are essential for subsequent treatment planning. In patients opting for breast conservation, thoroughly removing all gross tumors is linked to improved local control rates [10]. If no residual disease is anticipated, further surgery would be unnecessary. If a small amount of residual disease exists, the patient may benefit from re-excision. In cases of significant residual disease, a more individualized approach may be warranted, and mastectomy could be considered based on clinical judgment and patient factors [11].

Multiple studies have indicated that breast MRI is also valuable in assessing the presence or absence of residual cancer in patients after excisional biopsy with positive resection margins, undetermined margin status, or calcification-only lesions [12,13,14,15,16]. Even when breast cancer is detected post vacuum-assisted breast biopsy (VABB), breast MRI has proven to be highly sensitive and bears a high negative predictive value (NPV) in identifying residual breast tumors [17]. However, most studies have focused on the morphological analysis of postoperative sites [12,14,15,16], with only one including the kinetic evaluation of breast MRI to predict residual disease [13]. Furthermore, traditional imaging modalities such as mammography (MG) and ultrasound (US) remain standard practices, but their diagnostic performance in the context of residual cancer assessment has not been thoroughly evaluated or compared with MRI. Consequently, the effectiveness of these modalities in detecting residual disease is not well understood.

On the other hand, Schmidt et al. compared the ability of MG, US, and clinical examinations (palpation) to determine the residual size of breast cancer after neoadjuvant chemotherapy. Their study found that both MG and US were superior to palpation in predicting residual tumor size. However, neither method was able to predict pathological complete response or tumor margins with high confidence [18].

More recently, a study has highlighted the potential of DCE-MRI in preoperative chemotherapy monitoring, particularly in triple-negative breast cancer patients. The findings suggest that DCE-MRI can assess tumor responsiveness to chemotherapy and more accurately predict pathological response and prognosis. Kinetic parameters such as the washout index were found to correlate with residual tumor burden, and the absence of a fast-washout curve type in residual disease was identified as a significant prognostic factor as early as the midpoint of preoperative chemotherapy [19]. Additionally, ultrafast DCE-MRI using an empirical mathematical model has shown promise, with the initial slope of the kinetic curve correlating significantly with microvessel density in invasive breast cancer [20].

Recent advancements in imaging technology, such as diffusion-weighted imaging (DWI), present a promising complement to traditional CE-MRI, further improving diagnostic accuracy. DWI measures the diffusion of water molecules within tissues and is quantified by the apparent diffusion coefficient (ADC). Studies over the past decade have consistently shown that malignant breast lesions exhibit lower ADC values compared with benign lesions, suggesting that integrating DWI into breast MRI protocols could enhance diagnostic precision and specificity [21,22,23,24].

Given the growing interest in utilizing cutting-edge MRI techniques to improve breast cancer diagnosis and personalized treatment, our study aims to evaluate the efficacy of breast MRI, including DWI, in assessing residual lesions in patients diagnosed with malignancy after excisional biopsy. The goal is to improve diagnostic accuracy and guide optimal treatment strategies.

## 2. Materials and Methods

### 2.1. Study Population

**Figure 1:**
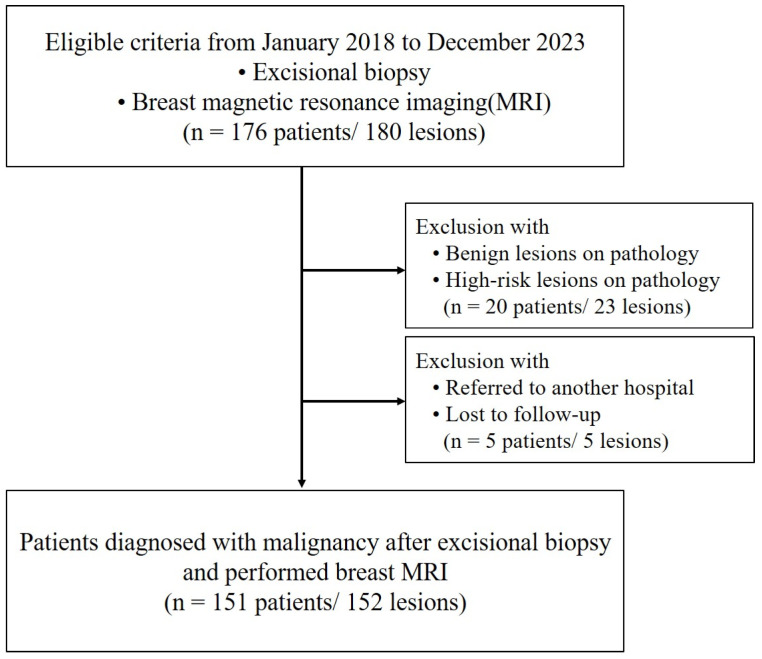
Flowchart of the study participants.

This study was conducted in accordance with the Declaration of Helsinki and was approved by our Institutional Review Board (IRB No. KC24RASI0539), and informed consent was waived owing to its retrospective nature. From January 2018 to December 2023, patients were eligible for inclusion if they had undergone an excisional biopsy and an MRI. Initially, 176 patients with 180 lesions were considered. However, patients with benign or high-risk lesions on pathology were excluded, reducing the cohort by 20 patients with 23 lesions. Moreover, patients who were referred to another hospital or lost to follow-up were also excluded, further reducing the cohort by 5 patients with 5 lesions. All patients underwent at least two rounds of follow-ups. Ultimately, the study included 151 patients with 152 lesions who were diagnosed with malignancy following excisional biopsy and had undergone breast MRI (Figure 1). Most of our patients underwent excisional biopsy at an outside hospital, so information on margin status was not available and, thus, was not included in the inclusion criteria.

### 2.2. Clinical and Histopathology Review

Clinical information for the enrolled cases was obtained from electronic medical records and included patient age, date of excisional biopsy, and the biopsy method. Surgical excisional biopsies were performed in 84.2% of lesions, while 15.8% were excised using vacuum-assisted techniques. Additionally, we collected data concerning clinicians’ treatment decisions, both surgical and non-surgical. For cases managed non-surgically, we reviewed the alternative treatment options considered.

Pathologic data were reviewed retrospectively. All excisional biopsy specimens gathered during the referral process were re-examined, and histology slides were reassessed to verify the initial diagnosis. After surgery, the specimens underwent evaluation for the date of surgery, type of surgery (including breast and axillary procedures), surgical site, tumor margin status, as well as the location and size of any residual disease, based on histopathologic assessment, and the presence of lymph node metastasis.

### 2.3. MRI Protocols

Breast MRI was performed using 3T machines (Verio and Vida, Siemens Healthcare, Erlangen, Germany). Patients were scanned in the prone position using a dedicated breast surface coil. The imaging protocol for both machines included axial T2-weighted imaging, diffusion-weighted imaging (DWI), and T1-weighted dynamic contrast-enhancement (CE) imaging. The Verio scanner utilized a turbo spin-echo sequence for axial T2-weighted imaging with a repetition time (TR) of 4530 ms and an echo time (TE) of 93 ms, featuring a flip angle of 80 degrees and incorporating 34 slices with a 4 mm thickness. DWI was performed using readout-segmented echoplanar imaging with *b*-values of 0 and 1000 s/mm^2^, and a TR/TE of 5200/53 ms. T1-weighted dynamic imaging was conducted using 3D volumetric interpolated breath-hold examination (VIBE) sequences both before and at multiple time points (10, 70, 130, 190, 250, and 310 s) following gadolinium DTPA injection (0.1 mmol/kg Gadovist). The Vida scanner employed a turbo spin-echo DIXON sequence for T2-weighted imaging with a TR/TE of 5000/96 ms and a flip angle of 120 degrees, accommodating 50 slices at a 3 mm thickness. DWI on this device used long variable echo trains, while T1-weighted dynamic imaging was executed with 3D fast low-angle shot (FLASH) sequences, before and at intervals (10, 93, 176, 259, 342, and 425 s) post-gadolinium injection. The Digital Imaging and Communications in Medicine (DICOM) files from DCE-MRI and DWI were processed using Syngovia software (version SOMARIS/8 VB70, Siemens Healthcare, Erlangen, Germany) to create maximum intensity projection (MIP) images and first post-contrast subtracted images.

### 2.4. MRI Analysis

MRI scans were retrospectively reviewed by two breast radiologists with 13 and 20 years of experience, respectively (H.S.M. and B.J.K.), who reached a consensus. The analysis adhered to the guidelines of the American College of Radiology Breast Imaging Reporting and Data System (BI-RADS). Fibroglandular tissue was classified as either non-dense or dense. Background parenchymal enhancement was evaluated and categorized as minimal to mild or moderate to marked. Lesion morphology was assessed through visual inspection of the contrast-enhanced subtraction images and was categorized into four types: no or minimal enhancement, linear and thin contrast enhancement (<2 mm) at the cavity border, thick or irregular enhancement at the cavity edge, and nodular or non-mass enhancement near the postoperative site [13,15,16]. The size of residual lesions was estimated, ranging from 0 (indicating no suspicious residual lesion) to the maximum diameter observed. Enhancement kinetics were examined based on patterns in the area of the most intense or uniform enhancement or in areas with visible residual enhancement. These patterns were identified as unassessable (where no residual lesion could be evaluated or minimal lesions could not be assessed for kinetics), delayed persistent or plateau, and delayed washout. Following the CE-MRI results, a diagnosis regarding the presence of residual lesions was determined as either ‘Absent’ or ‘Present’.

Additionally, ADC values were obtained by placing a small region of interest (ROI) on the darkest part of the lesion on the ADC map, deliberately avoiding cystic, noisy, or non-enhancing voxels. The diagnosis was subsequently refined by incorporating information from DWI, resulting in a final assessment that combined CE-MRI and DWI data, classified as either ‘Absent’ or ‘Present’. The radiologists also examined the axillary lymph nodes for metastasis, focusing on size and morphology.

### 2.5. Mammography/Ultrasound Interpretation

Out of the 152 cases included in the study, 67 cases with post-biopsy MG and US images were available for analysis. These images were retrospectively reviewed by two breast radiologists with 9 and 23 years of experience, respectively (G.E.P. and S.H.K.), who reached a consensus. The presence of residual lesions was independently assessed on both MG and US. For patients with post-biopsy MG, lesions were categorized into two groups: those involving calcifications, with or without an associated mass or asymmetry, and others, which included mass, asymmetry, or architectural distortion. MG findings were considered ‘Present’ if suspicious calcifications, a suspicious remnant mass, or asymmetry were noted adjacent to the excision site. US findings were classified as ‘Present’ if irregular margins with focal increased vascularity, a suspicious mass, or a non-mass lesion were detected around the postoperative site.

### 2.6. Statistical Analysis

Summary statistics are presented as counts (percentages) for categorical variables and as means (standard deviations) or medians (interquartile ranges) for continuous variables. The final results for residual malignancy were categorized as either present or absent, encompassing both surgical and non-surgical management approaches. To clarify, “Final results: present” refers to cases where a pathologically confirmed residual lesion was identified after surgery. In contrast, “Final results: absent” includes cases where no pathologically confirmed residual lesion was identified following surgery, as well as cases that were not surgically managed. MRI, MG, and US analyses were each compared separately with the final results. The comparisons utilized the Chi-square test or Fisher’s exact test for categorical variables and t-tests for continuous variables, respectively. Receiver Operating Characteristic (ROC) analysis was employed to assess the performance of each diagnostic method, based on the Area Under the Curve (AUC). We assumed a clinically meaningful difference with an expected AUC difference of 0.09. With a two-tailed alpha level of 0.05, our sample size of 152 cases provided 87.4% power. Performance metrics, including sensitivity, specificity, positive predictive value (PPV), and negative predictive value (NPV), were calculated and stratified according to the excisional biopsy to MRI time interval and the presence of a residual lesion. All statistical analyses were performed using SAS version 9.4 (SAS Institute, Cary, NC, USA) and MedCalc version 22 (MedCalc Software, Ostend, Belgium), with two-sided *p*-values < 0.05 considered statistically significant.

## 3. Results

### 3.1. Clinical and Pathologic Characteristics, Along with Final Treatment Results

The study examined a total of 152 lesions, with participants having an average age of 51 ± 11 years (Table 1). An histopathological analysis of the excisional biopsy specimens showed that 63.8% of the lesions were non-invasive, while 36.2% were invasive malignancies. The average time between the excisional biopsy and the subsequent MRI was 15.9 ± 9.4 days, with a median interval of 14 days (IQR = 9 to 20). Table S1 indicates that all non-invasive malignancies were classified as DCIS, with 50.5% categorized as grade 1 (low). Invasive ductal carcinoma was the most prevalent among invasive malignancies, detected in 30 out of 55 cases with specified grades, with 40.9% graded as 1 (well differentiated).

Regarding further treatment after excisional biopsy, 65.1% of patients underwent surgery, of which, 36.4% had breast-conserving surgery, 34.3% had breast-conserving surgery with sentinel lymph node biopsy (SLNB), and the remaining 29.3% underwent other surgical procedures. Non-surgical management was chosen by 34.9% of patients, among them, 84.9% received radiation therapy and 15.1% underwent surveillance with follow-up MRI. The mean interval from MRI to surgery was 14.7 ± 12.1 days, with a median of 11 days (IQR = 6 to 19). The final results, combining surgical and non-surgical management, indicated that 63.8% of lesions had no residual lesion after excisional biopsy, while 36.2% had residual malignancy. Surgically confirmed residual malignancies averaged 2.6 ± 3 cm in size, with a median of 1.2 cm (IQR = 0.4 to 4.2). Lymph node metastasis was present in 4% of cases, while 96.1% of patients showed no evidence of metastasis (Table 1).

### 3.2. Image Analysis Results

**Figure 2:**
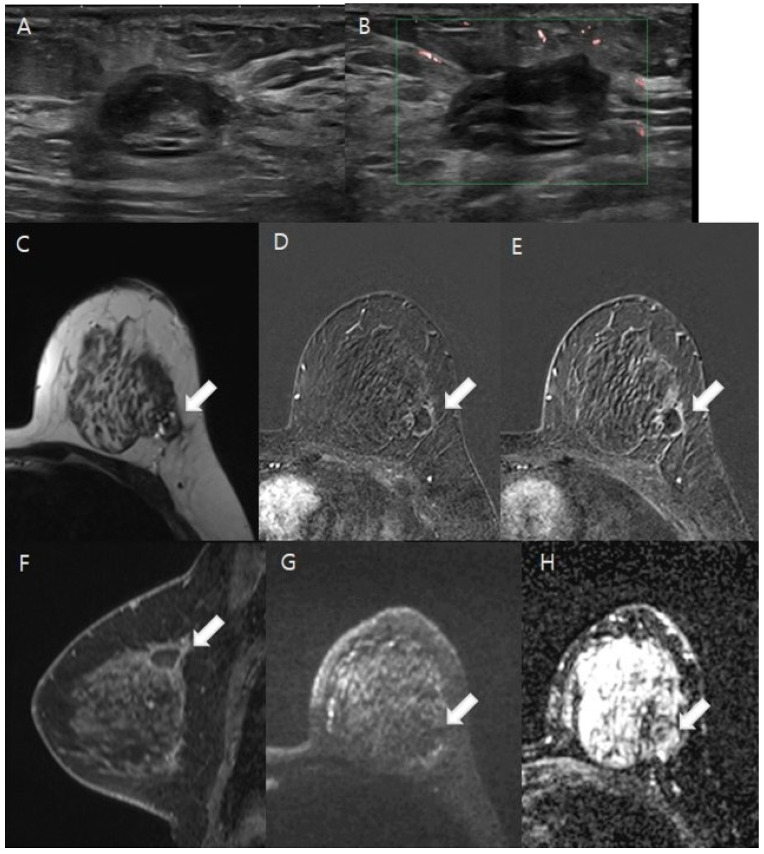
A 41-year-old woman diagnosed with ductal carcinoma in situ (DCIS) after an excisional biopsy. (**A**,**B**). The ultrasound reveals an oval hypoechoic lesion with internal hyperechogenicity located in the dependent area of the left upper outer quadrant of the breast. Doppler ultrasound showed no intra- or perilesional vascularity. (**C**–**H**). White arrows indicate the lesions in the following images. On T2-weighted imaging, the lesion presented heterogeneous high and low signal intensities, indicative of an organizing hematoma (**C**). Thin, linear marginal enhancement was apparent in post-contrast axial and sagittal images (**D**–**F**). Diffusion-weighted imaging (DWI) at b = 1000 mm^2^/s and the ADC map (b = 0 and 1000 mm^2^/s) demonstrated no diffusion restriction, suggesting the absence of residual malignancy (**G**,**H**). The patient then underwent breast-conserving surgery, and the pathology confirmed no residual malignancy in the left upper outer quadrant.

**Figure 3:**
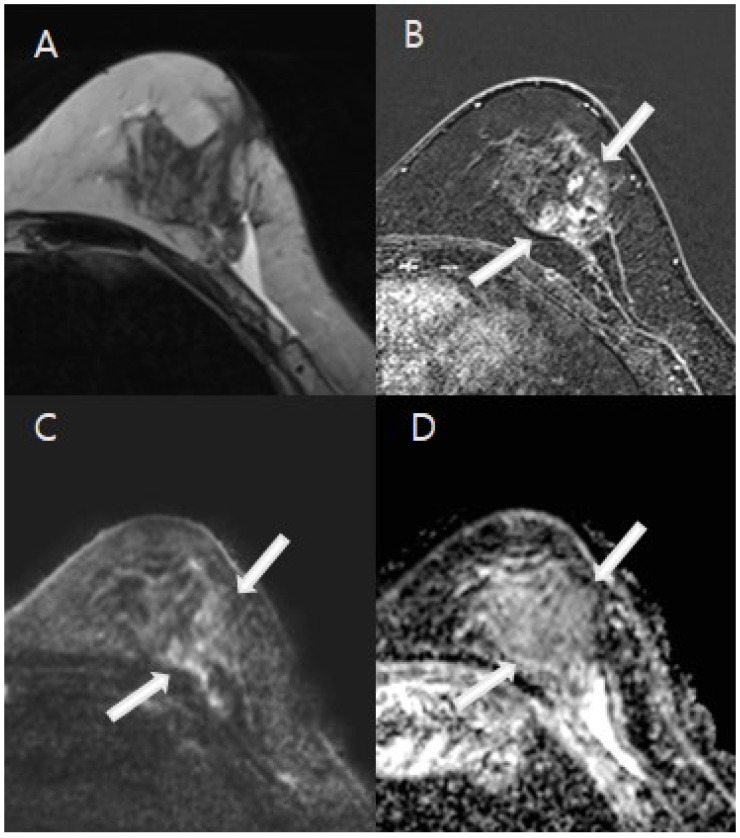
A 44-year-old woman with excisional biopsy-proven ductal carcinoma in situ (DCIS). (**A**). MRI demonstrates a parenchymal defect with T2 high signal intensity, suggesting fluid collection at the biopsy site in the mid outer aspect of the left breast. (**B**). A 2.8 cm area of regional nodular non-mass enhancement (white arrows) was noted in the medial aspect of the biopsy cavity. (**C**,**D**). Diffusion-weighted imaging (DWI) at b = 1000 mm^2^/s and the ADC map (b = 0 and 1000 mm^2^/s) indicated restricted diffusion in the corresponding area, with an ADC value of 1.3 × 10^−3^ mm^2^/s, indicative of residual malignancy (white arrows). The patient underwent a mastectomy, and final pathology confirmed a 3 cm area of DCIS.

**Figure 4:**
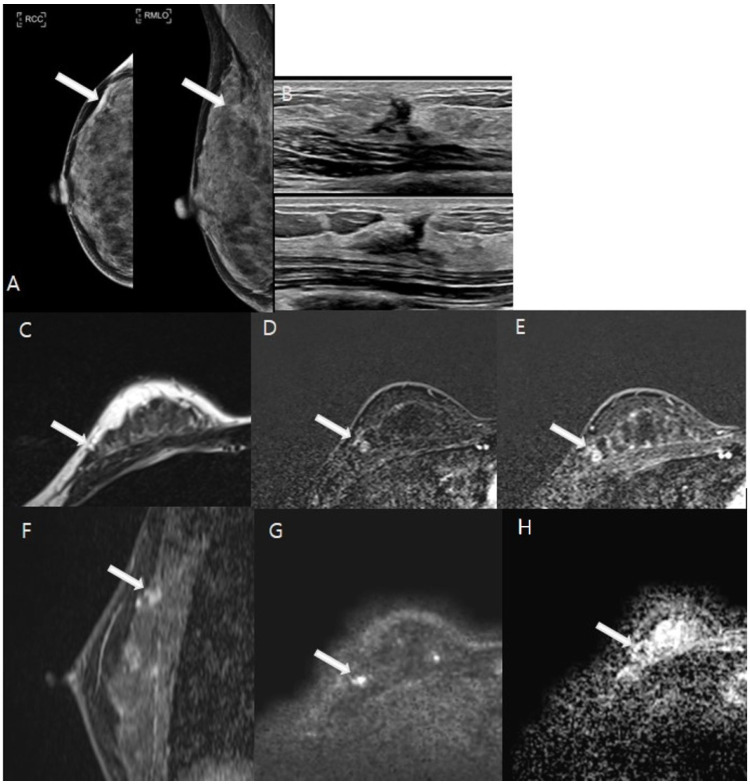
A 47-year-old woman underwent vacuum-assisted excisional biopsy and was diagnosed with ductal carcinoma in situ (DCIS). White arrows indicate the lesions in the following images. (**A**). Post-biopsy mammography showed architectural distortion with several round microcalcifications in the right upper outer quadrant. (**B**). Ultrasound revealed an irregular hypoechoic area corresponding to post-biopsy changes in the same region. (**C**–**H**). A 1 cm focal area of T2 isointense signal was observed adjacent to the post-biopsy changes (**C**). This area exhibited heterogeneous enhancement from the initial to the delayed phase on axial images (**D**,**E**), with enhanced visualization on sagittal images (**F**). Diffusion-weighted imaging (DWI) at b = 1000 mm^2^/s and the ADC map (b = 0 and 1000 mm^2^/s) indicated restricted diffusion within the lesion, with an ADC value of 0.9 × 10^−3^ mm^2^/s (**G**,**H**). The patient subsequently underwent breast-conserving surgery, which confirmed a residual 0.8 cm grade 1 DCIS on pathological examination.

The MRI analyses for diagnosing residual malignancies revealed several significant findings, as detailed in Table 2. Notably, there were substantial differences in lesion morphology (*p* < 0.001). Specifically, 59.8% of patients without residual malignancies demonstrated linear and thin contrast enhancement less than 2 mm at the cavity border (Figure 2), whereas 61.8% of patients with residual malignancies exhibited nodular or non-mass enhancement around the postoperative site (Figure 3). The predicted residual size also differed significantly (*p* < 0.001), with 80.4% of patients without residual malignancies having a predicted size of 1 cm or less. The kinetic patterns revealed significant differences (*p* < 0.001), with 95.9% of patients without residual malignancies displaying either delayed persistent or plateau kinetics, whereas 25.5% of those with residual malignancies demonstrated delayed washout, compared with only 1% of patients without residuals. Significant differences in ADC values were observed (*p* < 0.001). A value of ≥1.5 × 10^−3^ mm^2^/s was noted in 79.0% of patients without residual malignancies, while values <1.0 × 10^−3^ mm^2^/s were recorded in 16.4% of patients with residual malignancies (Figure 4). Both CE-MRI and CE-MRI with DWI demonstrated significant results (*p* < 0.001) in detecting residual malignancies, identifying 49.3% and 48.7% of cases, respectively. However, the MRI diagnosis of lymph node metastasis did not reach statistical significance (*p* = 0.069).

On MG, 43.3% of lesions were identified as containing calcifications, and 56.7% were classified as other types without calcifications. The difference in lesion type between patients with and without residual malignancies was not statistically significant (*p* = 0.074). The predictions of residual malignancies using MG and US approached but did not achieve statistical significance (*p* = 0.114 and *p* = 0.141, respectively) (Table S2).

### 3.3. The Diagnostic Performance of the Imaging Modalities

The diagnostic performance of the imaging modalities is summarized in Table 3. CE-MRI with DWI demonstrated a sensitivity of 90.9% and an NPV of 93.6%, compared with CE-MRI alone, which showed values of 89.1% and 92.2%, respectively. For MG, sensitivity was 57.1% and NPV was 64.7%, while for US, sensitivity was 38.7% and NPV was 59.6%. Diagnostic accuracy was 80.9%, 79.0%, 60.3%, and 59.7% for CE-MRI with DWI, CE-MRI alone, MG, and US, respectively.

For imaging within 14 days post-biopsy, CE-MRI with DWI achieved a sensitivity of 86.2% and an NPV of 89.7%, compared with CE-MRI alone at 82.8% and 87.2%. Imaging conducted after 14 days resulted in 100% sensitivity and NPVs for both MRI modalities.

In analyses based on residual lesion size, CE-MRI with DWI achieved a sensitivity of 76.2% and an NPV of 93.2% for detecting smaller lesions (≤1 cm), compared with CE-MRI alone, which had a sensitivity of 71.4% and an NPV of 92.0%. For larger lesions (>1 cm), both MRI modalities achieved 100% sensitivity and an NPV.

**Figure 5:**
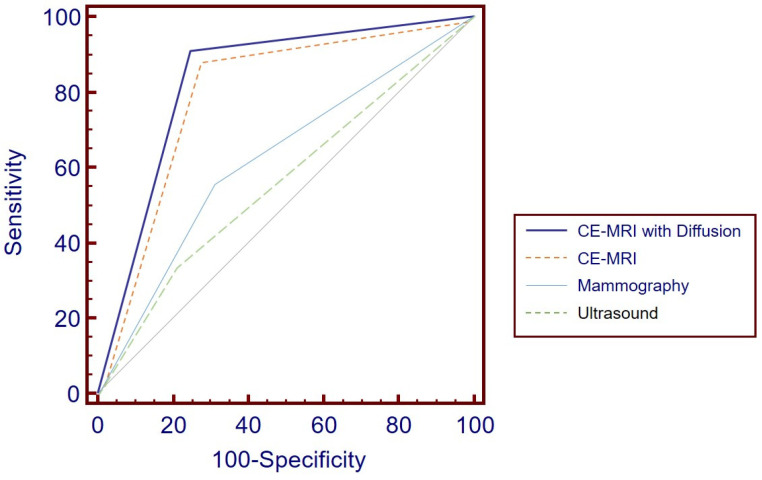
ROC Curve Analysis for Diagnostic Performance: CE-MRI with DWI achieved the highest AUC at 0.831 ± 0.030 (95% CI, 0.762 to 0.887), although this was not significantly different from CE-MRI alone, which had an AUC of 0.811 ± 0.031 (95% CI, 0.740 to 0.870) (*p* = 0.095). Mammography and ultrasound yielded lower AUCs of 0.623 ± 0.0655 (95% CI, 0.483 to 0.749) and 0.563 ± 0.060 (95% CI, 0.424 to 0.695), respectively. ROC = receiver operating characteristic, CE-MRI = contrast-enhanced MRI, DWI = diffusion-weighted imaging, AUC = area under the curve.

The ROC analysis compared the diagnostic performance, as illustrated in Figure 5. The AUC for CE-MRI with DWI was 0.831 ± 0.030 (95% CI, 0.762 to 0.887), while for CE-MRI alone, it was 0.811 ± 0.031 (95% CI, 0.740 to 0.870). Although CE-MRI with DWI exhibited a slightly higher AUC, suggesting a marginally better diagnostic performance, the difference between the two methods was not statistically significant (*p* = 0.095). For MG, the AUC was 0.623 ± 0.0655 (95% CI, 0.483 to 0.749), and for US, it was 0.563 ± 0.060 (95% CI, 0.424 to 0.695). Both MG and US demonstrated lower AUC values compared with the MRI modalities, with all comparisons being less than 0.05; however, there was no statistically significant difference between MG and US (*p* = 0.234).

## 4. Discussion

This study encompassed 151 patients with 152 lesions, all diagnosed with malignancy following excisional biopsy and who subsequently underwent breast MRI. Both CE-MRI and CE-MRI with DWI effectively identified residual malignancy, though this difference was not significant (*p* = 0.095). The diagnostic performance of MG and US was significantly lower than that of the MRI modalities.

In contrast to other studies [12,13,14,15,16,17], which involved populations confirmed through surgical interventions, our research uniquely illustrates the non-surgical management (34.9%) present in real clinical practice. Notably, there were no significant differences in age between the non-surgically and surgically managed groups (51 ± 9 years vs. 51 ± 12 years, *p* = 0.795). However, a majority of the non-surgical cases exhibited non-invasive pathology on excisional biopsy (50 out of 53 cases vs. 46 out of 99 cases, *p* < 0.001). The final results showed that 36.2% of all patients had residual malignancy. When focusing only on cases that underwent surgery, the rate of residual malignancy was 56% (55/99), aligning with ranges reported in previous studies. The rate of residual disease at histopathologic examination after definitive surgery has been reported to range from 33% to 70.9% [12,13,14,15].

MRI is more effective than conventional imaging techniques, such as MG and US [25], in assessing the preoperative extent of disease. This is especially evident in the postoperative breast, where postsurgical changes like architectural distortion and hematoma may either obscure or resemble malignancy [16]. Our study indicates that MG and US have limited predictive capabilities for residual malignancies following excisional biopsy. In support of this, a subset of patients underwent VABB followed by further surgery, revealing that MG, US, and MRI had detection accuracies for residual malignancy of 30.8%, 59.6%, and 61.2%, respectively [26]. Additionally, another study on microcalcifications showed that the average area under the AUC for predicting residual malignancy post-excisional biopsy was 0.601 (*p* = 0.231) for MG and 0.696 (*p* = 0.02) for MRI [12]. These findings suggest that MRI offers a more reliable assessment of residual disease, reinforcing its value in clinical practice.

Utilizing both morphologic and kinetic evaluations is crucial in breast MRI [27,28,29,30,31,32]. Invasive cancers typically exhibit rapid and pronounced contrast uptake followed by a washout curve in the delayed phase [28]. Incorporating these evaluations has been proven to improve diagnostic performance post-excisional biopsy [13]. Our study highlights the significance of certain MRI characteristics in predicting residual malignancies, including lesion morphology, estimated residual size, kinetic patterns, and ADC values. Although CE-MRI with DWI marginally outperformed CE-MRI alone in terms of AUC, the difference was not statistically significant, implying comparable diagnostic efficacy for both tests. In clinical practice, CE-MRI with DWI would enable clinicians to determine whether to proceed with additional surgery or adopt a more conservative approach. It could potentially reduce reoperation rates, minimize the risk of incomplete tumor removal, and improve patient outcomes.

DWI is evaluated alongside CE-MRI and indicates that lesions 6 mm or larger in the axial plane are assessable effectively. However, partial volume effects should be considered for smaller lesions [33], which may account for the lack of statistical significance. In five instances of pathologically confirmed residual malignancy, CE-MRI with DWI failed to detect the malignancy. One case presented challenges due to pronounced background parenchymal enhancement, while the remaining cases involved small residual malignancies measuring 0.2 cm, 0.3 cm, 0.5 cm, and 0.6 cm, as documented in the histopathologic review. These findings underscore the need for clinicians to consider the limitations of DWI, particularly for small lesions, while recognizing the overall superiority of MRI in assessing residual disease compared with MG and US.

In our research, the most prevalent pathologic diagnosis was ductal carcinoma in situ (62.5%). Previous studies reported rates of ductal carcinoma in situ ranging from 17% to 47.8% [13,16,34,35], which are lower than our findings. MRI sensitivity for detecting ductal carcinoma in situ varies widely (40–100%) and is generally lower than that for invasive ductal carcinoma [36,37]. Despite the higher incidence of ductal carcinoma in situ, MRI’s sensitivity for predicting residual disease in our study was consistent with prior reports.

There is no consensus on the optimal time interval between excisional biopsy and MRI. One previous study indicated that the highest specificity (75%) for evaluating residual disease was achieved between 28 and 35 days post-surgery, suggesting a minimum interval of 28 days before assessment [38]. In contrast, a subsequent study reported higher specificity and sensitivity when MRI was performed within 28 days of surgery [39]. A more recent study observed a trend of decreasing specificity and PPV as the interval between excision and breast MRI increased [13]. In our study, stratifying the data by a median of 14 days, CE-MRI with DWI yielded better diagnostic accuracy within the first 14 days post-biopsy, showing higher sensitivity and specificity compared with CE-MRI alone. Both modalities maintained excellent sensitivity and NPV across various time intervals, excelling particularly in early post-biopsy imaging. Thus, we also suggest that MRI assessments should not be unduly delayed following an excisional biopsy to avoid postponing definitive surgery.

Regarding lesion size, MRI measurements have been shown to correlate with pathological tumor size within a twofold range in 51 of 66 true-positive cases (77%) [13]. In our study, categorizing lesion size by a median of 1 cm, both techniques were effective in detecting larger lesions. However, CE-MRI with DWI demonstrated superior sensitivity and accuracy for smaller lesions. Overall, CE-MRI with DWI outperformed CE-MRI alone in detecting smaller lesions and provided better specificity for larger lesions, underscoring its enhanced diagnostic capabilities.

Our study has several limitations. First, our study was a retrospective analysis, and most patients underwent their excisional biopsies at outside hospitals. As a result, we lacked information on the initial characteristics of the breast lesions and the reasons for conducting the excisional biopsy, which could have possible implications for diagnosis and treatment. However, all excisional biopsy specimens gathered during the referral process were re-examined, and histology slides were reassessed to verify the initial diagnosis. Second, the reasons for choosing non-surgical management for patients were not clearly documented in the medical records. While we hypothesize that MRI diagnosis may influence treatment decisions, other factors—such as confirmed non-invasive cancers in excisional biopsy histology—could also have played a role. However, age alone did not appear to impact the decision-making process. Nonetheless, our study is the first to investigate the prevalence of non-surgical management in clinical practice. Third, our results indicate that the ability of MG and US to predict residual malignancies after excisional biopsy is limited, though further investigation is required to generalize these findings. In routine practice, patients underwent MG and US before excisional biopsy, and those with unexpected malignancies were referred for breast MRI. As a result, it is assumed that the interpretation of MG and US images post-excisional biopsy was applicable only to selected cases. Finally, all study participants underwent at least two rounds of follow-up. To date, only one recurrent case has been detected; this patient, who had undergone radiation therapy, experienced a recurrence at the excisional biopsy site after two years. Continued surveillance is necessary to fully assess long-term outcomes.

This study contributes significant new knowledge regarding the effectiveness of breast MRI in detecting residual malignancies following excisional biopsy, particularly the advantages of combining CE-MRI with DWI. Our findings show that MRI assessments conducted within 14 days post-biopsy yield an 84.5% diagnostic accuracy for detecting residual disease, which is critical for making informed clinical decisions. By demonstrating that CE-MRI with DWI is particularly effective in identifying smaller lesions, with a negative predictive value of 93.2%, our research emphasizes the importance of prioritizing MRI evaluations in clinical practice. This timely approach not only aids in better patient management but also facilitates appropriate treatment planning, ultimately improving patient outcomes in breast cancer care.

In conclusion, breast MRI is effective in detecting residual malignancies after excisional biopsy. Both CE-MRI with DWI and CE-MRI alone demonstrated comparable, excellent performance in discriminating between women with and without residual disease. Therefore, this study suggests that the combination of CE-MRI and DWI has the potential to become a standard imaging protocol for patients with suspected residual tumors after excisional biopsy.

## Figures and Tables

**Table 1:** Clinical and pathologic characteristics, along with final results, of total lesions (n = 152).

	Total
	n = 152
Age (years)	
Mean ± SD	51 ± 11
Median (IQR)	49 (44, 57)
Excisional biopsy pathology	
Non-invasive	97 (63.8)
Invasive	55 (36.2)
Excisional biopsy to MRI time interval (days)	
Mean ± SD	15.9 ± 9.4
Median (IQR)	14 (9, 20)
Treatment options	
Surgery (n = 99, 65.1%)	
Breast-conserving surgery	36 (36.4)
Breast-conserving surgery with SLNB	34 (34.3)
Mastectomy only	1 (1)
Mastectomy with SLNB	16 (16.2)
SLNB only	12 (12.1)
Non-surgical management (n = 53, 34.9%)	
Radiation therapy	45 (84.9)
Surveillance with follow-up MRI	8 (15.1)
MRI to surgery time interval (days)	
Mean ± SD	14.7 ± 12.1
Median (IQR)	11 (6, 19)
Final results for presence of residual lesion	
Absent	97 (63.8)
Present	55 (36.2)
Final residual lesion size (cm)	
Mean ± SD	2.6 ± 3
Median (IQR)	1.2 (0.4, 4.2)
Final diagnosis of lymph node metastasis	
Absent	146 (96.1)
Present	6 (4)

Values are numbers (percentages) for categorical variables, and means (SD) or medians (IQR) for continuous variables. SD = standard deviation, IQR = interquartile range, SLNB = sentinel lymph node biopsy.

**Table 2:** MRI analysis in assessing residual lesions after excisional biopsy compared with final results.

	Total	Absent	Present	
	n = 152	n = 97	n = 55	*p*-Value
Fibroglandular tissue				0.435
Non-dense	27 (17.8)	19 (19.6)	8 (14.6)	
Dense	125 (82.2)	78 (80.4)	47 (85.5)	
BPE				0.764
Minimal to mild	86 (56.6)	54 (55.7)	32 (58.2)	
Moderate to marked	66 (43.4)	43 (44.3)	23 (41.8)	
Morphology of enhancement				<0.001
No or minimal	11 (7.2)	8 (8.3)	3 (5.5)	
Linear and thin (<2 mm)	59 (38.8)	58 (59.8)	1 (1.8)	
Thick or irregular	36 (23.7)	19 (19.6)	17 (30.9)	
Nodular or non-mass	46 (30.3)	12 (12.4)	34 (61.8)	
Predicted residual size (cm)				<0.001
Mean ± SD	1.5 ± 2.2	0.6 ± 1.4	2.9 ± 2.7	
Median (IQR)	0.6 (0, 1.6)	0 (0, 0.8)	1.5 (0.8, 5.1)	
Categorized into groups				<0.001
≤1 cm	99 (65.1)	78 (80.4)	21 (38.2)	
>1 cm	53 (34.9)	19 (19.6)	34 (61.8)	
Kinetics				<0.001
Unassessable	7 (4.6)	3 (3.1)	4 (7.3)	
Delayed persistent/plateau	130 (85.5)	93 (95.9)	37 (67.3)	
Delayed washout	15 (9.9)	1 (1)	14 (25.5)	
ADC value (mm^2^/s)				<0.001
≥1.5 × 10^−3^ or unassessable	120 (79)	95 (97.9)	25 (45.5)	
1.0 to <1.5 × 10^−3^	23 (15.1)	2 (2.1)	21 (38.2)	
<1.0 × 10^−3^	9 (5.9)	-	9 (16.4)	
CE- MRI diagnosis				<0.001
Absent	77 (50.7)	71 (73.2)	6 (10.9)	
Present	75 (49.3)	26 (26.8)	49 (89.1)	
CE-MRI with DWI diagnosis				<0.001
Absent	78 (51.3)	73 (75.3)	5 (9.1)	
Present	74 (48.7)	24 (24.7)	50 (90.9)	
MRI for LN metastasis				0.069
Absent	134 (88.2)	89 (91.8)	45 (81.8)	
Present	18 (11.8)	8 (8.3)	10 (18.2)	

Values are numbers (percentages) for categorical variables, and means (SD) or medians (IQR) for continuous variables. *p* values are calculated using the Chi-square test or Fisher’s exact test for categorical variables, and the t-test for continuous variables. SD = standard deviation, IQR = interquartile range, BPE = background parenchymal enhancement, CE-MRI = contrast-enhanced MRI, DWI = diffusion-weighted imaging, LN = lymph node.

**Table 3:** Diagnostic Performance for Presence of Residual Lesion by Mammography, Ultrasound, and MRI: Subgroup Analysis Based on Excisional Biopsy to MRI Time Interval and Residual Lesion Size.

	Sensitivity	Specificity	PPV	NPV	Accuracy
	(95% CI)	(95% CI)	(95% CI)	(95% CI)	(95% CI)
MG	57.1	62.9	55.2	64.7	60.3
	(37.2–75.5)	(44.9–78.5)	(35.7–73.6)	(46.5–80.3)	(47.2–72.4)
US	38.7	77.8	60.0	59.6	59.7
	(21.9–57.8)	(60.9–89.9)	(36.1–80.9)	(44.3–73.6)	(47.0–71.5)
CE-MRI	89.1	73.2	65.3	92.2	79.0
	(77.8–95.9)	(63.2–81.7)	(53.5–76.0)	(83.8–97.1)	(71.6–85.1)
CE-MRI with DWI	90.9	75.3	67.6	93.6	80.9
	(80.1–97.0)	(65.5–83.5)	(55.7–78.0)	(85.7–97.9)	(73.8–86.8)
≤14 days					
CE-MRI	82.8	81.0	75.0	87.2	81.7
	(64.2–94.2)	(65.9–91.4)	(56.6–88.5)	(72.6–95.7)	(70.7–89.9)
CE-MRI with DWI	86.2	83.3	78.1	89.7	84.5
	(68.3–96.1)	(68.6–93.0)	(60.0–90.7)	(75.8–97.1)	(74.0–92.0)
>14 days					
CE-MRI	100	66.7	52.8	100	75.7
	(82.4–100)	(52.1–79.2)	(35.5–69.6)	(89.7–100)	(64.0–85.2)
CE-MRI with DWI	100	68.6	54.3	100	77.1
	(82.4–100)	(54.1–80.9)	(36.7–71.2)	(90.0–100)	(65.7–86.3)
≤1 cm					
CE-MRI	71.4	88.5	62.5	92.0	84.9
	(47.8–88.7)	(79.2–94.6)	(40.6–81.2)	(83.4–97.0)	(76.2–91.3)
CE-MRI with DWI	76.2	88.5	64.0	93.2	85.9
	(52.8–91.8)	(79.2–94.6)	(42.5–82.0)	(84.9–97.8)	(77.4–92.1)
>1cm					
CE-MRI	100	10.5	66.7	100	67.9
	(89.7–100)	(1.3–33.1)	(52.1–79.2)	(15.8–100)	(53.7–80.1)
CE-MRI with DWI	100	21.1	69.4	100	71.7
	(89.7–100)	(6.1–45.6)	(54.6–81.8)	(39.8–100)	(57.7–83.2)

CI = confidence interval, PPV = positive predictive value, NPV = negative predictive value, MG = mammography, US = ultrasound, CE-MRI = contrast-enhanced MRI, DWI = diffusion-weighted imaging.

## Data Availability

The datasets used and/or analyzed during the current study are available from the corresponding author on reasonable request.

## Supplementary Materials

The following supporting information can be downloaded at: https://www.mdpi.com/article/10.3390/tomography11010010/s1, Table S1: Histopathological analysis by excisional biopsy; Table S2: Lesion characteristics on mammography and ultrasound after excisional biopsy compared with final results.
