# Supplementary material for: Enhanced Detection of Residual Breast Cancer Post-Excisional Biopsy: Comparative Analysis of Contrast-Enhanced MRI with and Without Diffusion-Weighted Imaging
Source: Tomography. 2025 Jan 20;11(1):10. doi: 10.3390/tomography11010010 (PMC11769435; doi:10.3390/tomography11010010)
Supplement: Supplementary file 1 [file tomography-11-00010-s001.zip › tomography-3388761-supplementary.pdf]

Table S1. Histopathological Analysis by Excisional Biopsy

	Total<br>n=152
**Tumor Histology**	
DCIS	95 (62.5)
Invasive Ductal Carcinoma	30 (19.7)
Mucinous Carcinoma	5 (3.3)
Papillary Carcinoma	4 (2.6)
Malignant Phyllodes Tumor	5 (3.3)
Others	13 (8.6)
**DCIS Nuclear Grade** (n=97)	
Grade 1 (Low)	49 (50.5)
Grade 2 (Intermediate)	35 (36.1)
Grade 3 (High)	13 (13.4)
**Invasive Tumor Grade** (n=44)*	
Grade 1 (Well Differentiated)	18 (40.9)
Grade 2 (Moderately Differentiated)	14 (31.8)
Grade 3 (Poorly Differentiated)	12 (27.3)
**Estrogen Receptor Status**	
Absent (-)	18 (12.4)
Present (+)	127 (87.6)
**Progesterone Receptor Status**	
Absent (-)	22 (15.5)
Present (+)	120 (84.5)
**HER2 Status**	
Absent (-)	123 (87.2)
Present (+)	18 (12.8)
**Ki-67 Index**	
≤20%	112 (80)
>20%	28 (20)

**Tumor Subtype**	
HR+/HER2-	113 (80.1)
HR+/HER2+	10 (7.1)
HR-/HER2+	8 (5.7)
HR-/HER2-	10 (7.1)

Values are numbers (percentages) for categorical variables and means (SD), median (IQR) for continuous variables.

*Fifty-five cases were confirmed to have invasive malignancies. However, some lesions, such as malignant phyllodes tumor, papillary carcinoma, and metaplastic carcinoma, were not assigned a specific grade in the pathology reports.

DCIS= ductal carcinoma in situ, HER2=epidermal growth factor receptor 2, HR=hormone receptor

Table S2. Lesion characteristics on mammography and ultrasound after excisional biopsy compared with final results (n=67)*

	Total	Absent	Present	P-Value
**Lesion Type**				0.074
Calcification With/Without Mass or Asymmetry	29 (43.3)	12 (32.4)	17 (56.7)	
Others (mass, asymmetry, or architectural distortion)	38 (56.7)	25 (67.6)	13 (43.3)	
**Mammography Diagnosis**				0.114
Absent	36 (53.7)	23 (62.2)	13 (43.3)	
Present	31 (46.3)	14 (37.8)	17 (56.7)	
**Ultrasound Diagnosis**				0.141
Absent	47 (70.2)	28 (77.8)	19 (61.3)	
Present	20 (29.9)	8 (22.2)	12 (38.7)	

Values are numbers (percentages) for categorical variables and means (SD), median (IQR) for continuous variables.

P values are calculated using the Chi-square test or Fisher's exact test for categorical variables and the t-test for continuous variables.

*Out of the 152 cases included in the study, 67 were referred to our tertiary hospital with their post-biopsy mammography and ultrasound images.
